# Distribution of Pediatric Vital Signs in the Emergency Department: A Nationwide Study

**DOI:** 10.3390/children7080089

**Published:** 2020-08-05

**Authors:** Woori Bae, Kyunghoon Kim, Bongjin Lee

**Affiliations:** 1 Department of Pediatrics, College of Medicine, The Catholic University of Korea, Seoul 06591, Korea; baewool7777@hanmail.net (W.B.); journey237@catholic.ac.kr (K.K.); 2 Department of Emergency Medicine, Seoul National University Hospital, Seoul 03080, Korea; 3 Department of Biomedical Engineering, Seoul National University College of Medicine, Seoul 03080, Korea

**Keywords:** child, heart rate, respiratory rate, vital signs

## Abstract

To effectively use vital signs as indicators in children, the magnitude of deviation from expected vital sign distribution should be determined. The purpose of this study is to derive age-specific centile charts for the heart rate and respiratory rate of the children who visited the emergency department. This study used the Korea’s National Emergency Department Information System dataset. Patients aged <16 years visiting the emergency department between 1 January 2016 and 31 December 2017 were included. Heart rate and respiratory rate centile charts were derived from the population with normal body temperature (36 to <38 °C). Of 1,901,816 data points retrieved from the database, 1,454,372 sets of heart rates and 1,458,791 sets of respiratory rates were used to derive centile charts. Age-specific centile charts and curves of heart rates and respiratory rates showed a decline in heart rate and respiratory rate from birth to early adolescence. There were substantial discrepancies in the reference ranges of Advanced Paediatric Life Support and Pediatric Advanced Life Support guidelines. Age-based heart rate and respiratory rate centile charts at normal body temperature, derived from children visiting emergency departments, serve as new evidence-based data and can be used in follow-up studies to improve clinical care for children.

## 1. Introduction

Vital signs reflect a patient’s fundamental physical status. They are used to detect and evaluate individuals who are in need of resuscitation, and to identify patients whose conditions are worsening, in the form of an early warning system [1,2,3]. To effectively utilize vital signs as indicators in children, it may be useful to identify the magnitude of deviation from the expected vital sign distribution and consider the child’s age and location of care (e.g., ED or general wards) rather than only to determine whether the vital sign values are abnormal. 

In the emergency department (ED), vital signs are used to assess the patient’s state, to triage the category of the patient, and to determine the disposition of patients and required resources [4]. All or most children who visit EDs have acute illnesses and various situations at EDs, such as unfamiliar people and place or an unpleasant experience with an injection, could easily influence a child’s vital signs. Typically, however, existing reference ranges for heart rate (HR) and respiratory rate (RR) in children were determined from children in outpatient clinics, healthy children in schools, or sleeping children in hospitals [5]. We believe that there are substantial differences in the HR and RR ranges of children who visit EDs.

There are a few studies that derived the reference range for HR and RR in children who visited EDs [6,7]. However, they have limitations because they were conducted in a single center or had the possibility to be confounded by the body temperature (BT) of the patients. We therefore conducted an investigation to derive age-based centile curves of HR and RR from the pediatric population visiting EDs in Korea and to compare these centiles with other reference ranges. 

## 2. Materials and Methods

### 2.1. Data Source

The data used in this study were provided by Korea’s National Emergency Department Information System (NEDIS), operated by the National Emergency Medical Center. Data were collected from 408 EDs (31 district emergency medical centers, 120 regional emergency medical centers, and 257 regional emergency medical departments) in 2016 and from 413 EDs (36 district emergency medical centers, 118 regional emergency medical centers, and 259 regional emergency medical departments) in 2017.

### 2.2. Inclusion and Exclusion Criteria

We included patients under 16 years of age who visited EDs with normal BT between 1 January 2016 and 31 December 2017. The patients who died at the EDs were excluded. In this study, the normal BT range was defined as 36 to <38 °C.

### 2.3. Data Collection and Pre-Processing

In the data abstraction process; age, HR, RR, and BT were collected for analysis. The following non-physiological values were removed to eliminate outliers that were thought to suggest errors in the data input process: HR > 300 beats/min, HR < 30 beats/min, RR > 120 breaths/min, and RR < 5 breaths/min.

### 2.4. Outcomes

The primary outcome was to derive age-based centile curves for HR and RR measured at normal BT in the children who visited EDs. The secondary outcome was the comparison of our data with the thresholds of Advanced Paediatric Life Support (APLS) [2] and Pediatric Advanced Life Support (PALS) [1] guidelines and with centile charts reported in previous studies [5,6,8].

### 2.5. Data Analysis

Box-Cox power exponential distribution and Lambda-Mu-Sigma methods were used to construct age-based centile charts for HR and RR, and the penalized B-spline method was used for smoothing. These processes were performed using the generalized additive model for location, scale, and shape package of R software [9,10,11,12,13]. Because a representative value of age is needed to display a dot in the graph, the graph uses the median value of the corresponding age range as the representative value. For example, those who were 3 to <4 years old were categorized as 3.5 years old. All data processing was performed using R version 3.6.1 (R Foundation for Statistical Computing, Vienna, Austria).

### 2.6. Ethics Statement

This study protocol was reviewed by the Institutional Review Board of Seoul National University Hospital and was exempted from deliberation because of the use of deidentified datasets from the NEDIS (E-1909-097-1065).

## 3. Results

### 3.1. Baseline Characteristics

**Figure 1:**
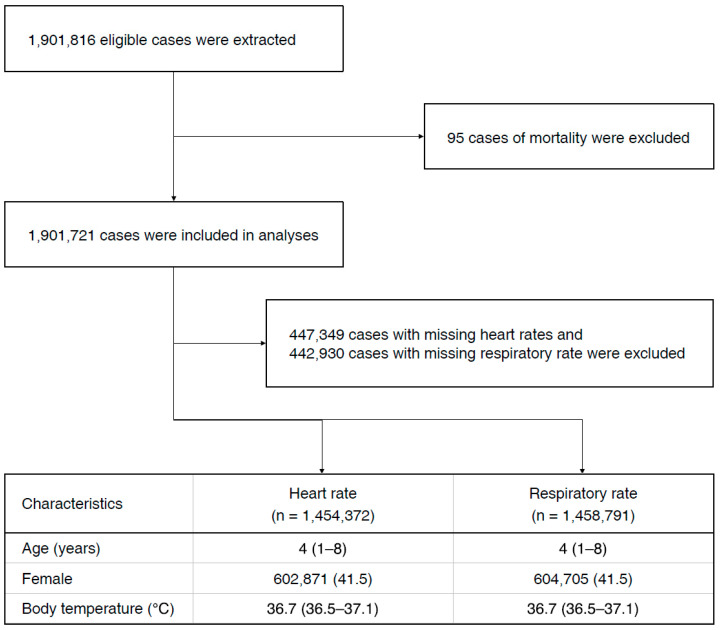
Flowchart of the screening process and the demographic data. Data are presented as median (interquartile range) or number (percentage) of participants.

After applying inclusion criteria and exclusion criteria, 1,901,721 cases were included in the analyses. Among them, 1,454,372 HR and 1,458,791 RR, excluding missing values, were used to derive centile charts. The flowchart and demographic characteristics of the patients are shown in Figure 1.

### 3.2. Primary Outcome

**Figure 2:**
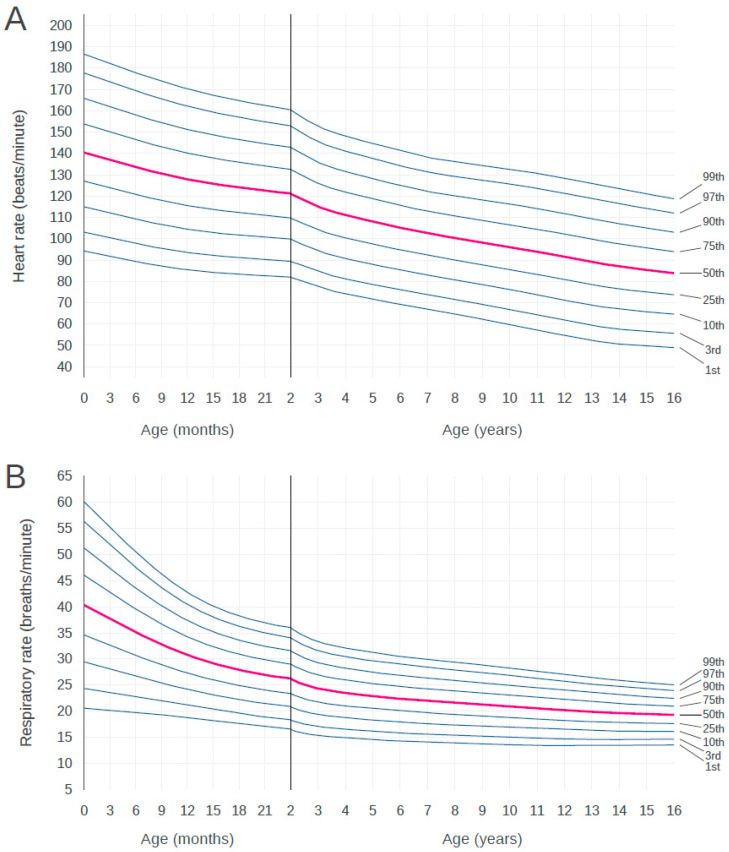
Centile curves for heart rate and respiratory rate by age. Centile curves for (**A**) heart rate and (**B**) respiratory rate by age in children with normal body temperature. The solid vertical line at 2 year of age represents a change in the scale of the x-axis.

Figure 2 shows the 1st to 99th centiles of HR and RR at normal BT in our cohort of birth to 16-year-old children visiting EDs during the 2-year sample period. These centiles show a decline in HR and RR from birth to early adolescence. In RR, the steepest decline is apparent in infants during the first 2 years of life. The centile charts of HR and RR by age are shown in Table 1 and Table 2, respectively. Age was divided into 3-month increments up to 24 months and continued in 1-year increments thereafter.

### 3.3. Secondary Outcomes

**Figure 3:**
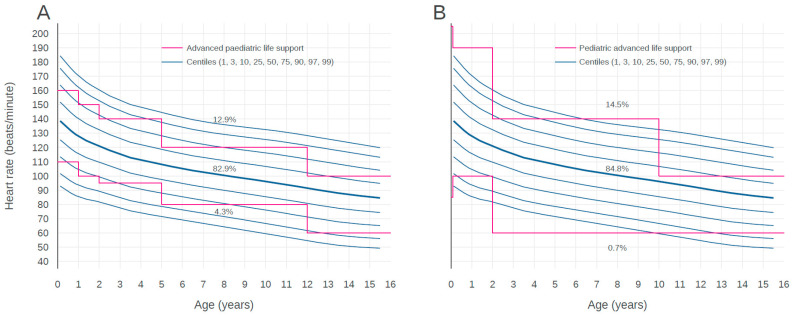
Centile curves for heart rate by age. Centile curves of heart rate by age with visual comparison against the centile curves from advanced pediatric life support (**A**) and pediatric advanced life support (**B**) guidelines.

Figure 3 shows a comparison of the reference ranges from the APLS and PALS guidelines with our centiles of HR. The HRs of 12.9% children who visited EDs were higher than the APLS upper limit and the HRs of 4.3% children were lower than the APLS lower limit. The HRs of 82.9% children existed within reference ranges of the APLS guideline. In the case of the PALS guideline, the HR of 14.5% children who visited EDs were higher than the PALS upper limit and the HRs of 0.7% children were lower than the PALS lower limit. The HRs of 84.8% children existed within the reference ranges of the PALS guideline. While the APLS upper and lower limits existed between the 1st and 99th centile curves across all age groups, the PALS upper limit was located outside of the 99th centile curves of children for ages up to 2 years and 6 to 10 years. The PALS lower limit was located outside of the 1st curves of children for ages 2 to 10 years.

**Figure 4:**
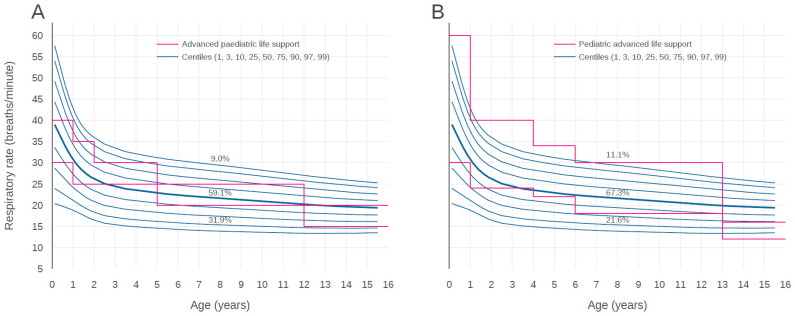
Centile curves for respiratory rate by age. Centile curves of respiratory rate by age with visual comparison against the centile curves from advanced pediatric life support (**A**) and pediatric advanced life support (**B**) guidelines.

Figure 4 shows the centile curves of RR according to age compared with the thresholds of both pediatric resuscitation guidelines. The RRs of 9.0% children who visited EDs were higher than the APLS upper limit and the RR of 31.9% children were lower than the APLS lower limit. The RRs of 59.1% children existed within the reference ranges of the APLS guideline. The RRs of 11.1% children was higher than PALS upper limit and the RRs of 21.6% children was lower than PALS lower limit. The RRs of 67.3% children existed within the reference ranges of the PALS guideline. While the APLS upper and lower limits existed between the 1st and 99th centile curves across all age groups, the PALS upper limit was located outside of the 99th centile curves of children for age up to 13 years. The PALS lower limit was located outside of 1st curves of children for age above 13 years.

**Figure 5:**
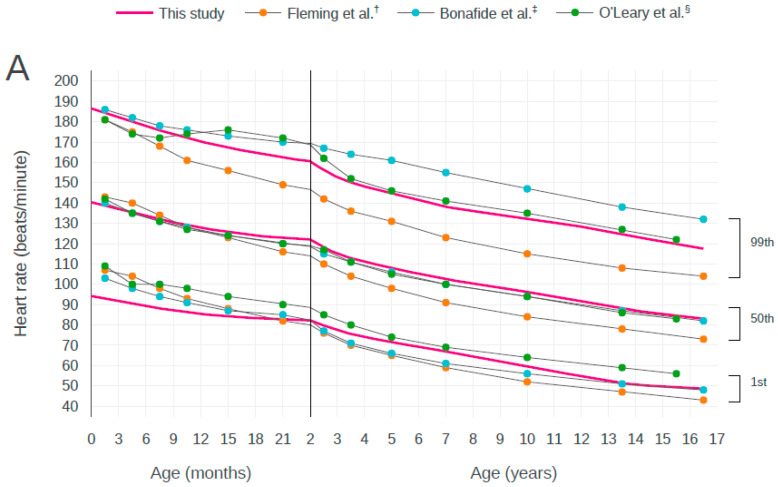

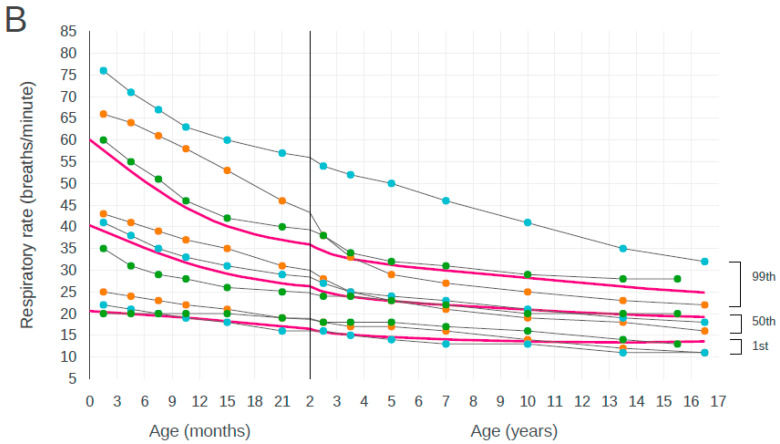
Comparison of the centile curves for heart rate and respiratory rate by age with the curves from previous studies. Centile curves of (**A**) heart rate and (**B**) respiratory rate by age with visual comparison against the centile curves from previous studies. The solid vertical line at 2 years of age represents a change in the scale of the *x*-axis. ^†^ Data from Fleming et al. [5]. ^‡^ Data from Bonafide et al. [8]. ^§^ Data from O’Leary et al. [6].

We compared our results with the centile curves reported in previous studies [5,6,8]. There were some differences between the centile curves, but there was general agreement that the centiles show a decline in HR and RR from birth to early adolescence. In curves for both HR and RR, the ranges indicated by Bonafide et al. (from the 1st centile to the 99th centile) were the widest for almost all ages (Figure 5).

The HRs of 12.9% children were higher than the APLS upper limit and the HRs of 4.3% of children were lower than the APLS lower limit. The HRs of 82.9% children existed within the reference ranges of APLS. The HRs of 14.5% children were higher than the PALS upper limit and the HRs of 0.7% children were lower than the PALS lower limit. The HRs of 84.8% children existed within the reference ranges of PALS.

The RRs of 9.0% children were higher than the APLS upper limit and the RRs of 31.9% children were lower than the APLS lower limit. The RRs of 59.1% children existed within the reference ranges of the APLS guidelines. The RRs of 11.1% children higher than the APLS upper limit and the RRs of 21.6% children were lower than the PALS lower limit. The RRs of 67.3% children existed within the reference ranges of the PALS guidelines.

## 4. Discussion

In this study, we derived distributions of HR and RR at normal BT and stratified by age in children who visited EDs in Korea, using data from a nationwide dataset, NEDIS. The ascertainment of reference ranges for vital signs in children remains challenging. To the best of our knowledge, this is the first study to derive specific distributions of HR and RR at normal BT in children in hospital ED settings throughout the country.

There have been prior attempts to derive age-based centile charts for children. Fleming et al. presented age-specific centiles developed for HR and RR by systematically reviewing 69 studies [5]. However, all referred studies were conducted among healthy or sleeping children. We know that sleeping children have lower HR and RR. We believe that there are substantial discrepancies in the ranges of HR and RR, because most children who visit EDs are awake at the EDs.

We derived distributions of HR and RR at normal BT in children who visited EDs. It is well known that HR and RR vary depending on BT [14,15,16,17]. HR and RR tended to increase with increasing BT at the time of measurement in our study. Therefore, the distribution of HR and RR should consider the BT of the population from which the distribution was derived. However, previous studies did not consider the effects of BT on HR and RR in children [5,8]. The reference range reported from a tertiary referral pediatric hospital in Australia excluded patients with BT above 38 °C [6]. This minimized the effects of high BT on other vital signs in their pediatric ED cohort. Nevertheless, the possibility of hypothermia remained.

APLS and PALS are widely used resources for the acute care of children. As shown in Figure 3 and Figure 4, the utility of thresholds recommended by APLS and PALS may be limited in our population. Graphical comparison suggests that the thresholds of both guidelines could lead to substantial under- or over-identification of at-risk children. We believe that the thresholds for HR and RR for pediatric resuscitation have substantial discrepancies with ranges obtained for pediatric populations in a real-world setting. This may be a natural consequence because the APLS and PALS guidelines have simplified thresholds in accordance with age for applying at the moment impending resuscitation. Further studies which compare the outcomes of assessed patients based on real-world data and simplified thresholds of resuscitation guidelines are needed to determine vital sign thresholds and to validate for setting alarm limits or risk-stratifying children.

This study has several limitations. First, this was a retrospective study which extracted the data from the registry. Since the data of this study were provided anonymously, the possibility that the HR or RR of the same patient being included more than once cannot be excluded. In addition, we could not control for all other factors, excluding fever, which could potentially affect the vital signs of children at Eds, such as pain, anxiety, and method of measurement. Second, to remove possible conditions that could affect HR or RR, we removed data of patients with cardiovascular disease, respiratory disease, and those who were admitted to the ICU. However, it was not possible to completely exclude more specific personal characteristics beyond those collected by the NEDIS. These are inherent limitations of de-identified public datasets and these are not specific to this study. Finally, there were some missing values of vital signs. However, this may explain the characteristics of pediatric patients who have difficulty in measuring vital signs compared to adults.

## 5. Conclusions

We derived pediatric centile curves for HR and RR by age under normal BT using a nationwide dataset from emergency medical institutions in Korea. We believe that our data provide a reliable representation of HR and RR distributions in children who visited EDs. These data could be used to establish alarm limits and risk-stratifying children in EDs. In addition, we believe our data can be used as baseline data for the comparing differences in HR and RR distribution in the pediatric population visiting EDs. Well-designed large prospective studies are needed to overcome the limitations of our study and to validate vital sign reference ranges in pediatric EDs.

## Figures and Tables

**Table 1:** Centile chart of heart rate per minute by age group.

Age	Centile												
	1st	3rd	5th	10th	15th	25th	50th	75th	85th	90th	95th	97th	99th
0–<3 months	93	102	106	113	118	125	139	152	159	164	171	176	184
3–<6 months	90	99	104	111	115	122	135	148	155	160	167	171	180
6–<9 months	88	97	101	108	112	119	132	145	151	156	163	167	176
9–<12 months	86	94	99	105	110	117	129	142	148	153	159	164	172
12–<15 months	85	93	97	104	108	115	127	139	145	150	156	161	169
15–<18 months	84	92	96	102	107	113	125	137	143	147	154	158	166
18–<21 months	83	91	95	101	105	112	123	135	141	145	152	156	164
21–<24 months	82	90	94	100	104	110	122	133	139	144	150	154	161
2–<3 years	80	87	91	97	101	107	118	129	135	139	145	149	156
3–<4 years	76	83	86	92	96	102	113	124	129	133	139	143	150
4–<5 years	73	80	84	89	93	99	110	120	126	130	136	139	146
5–<6 years	70	77	81	87	90	96	107	117	123	127	132	136	143
6–<7 years	68	75	79	84	88	93	104	114	120	123	129	133	140
7–<8 years	66	73	76	82	86	91	101	112	117	121	127	130	137
8–<9 years	64	70	74	80	83	89	99	110	115	119	125	128	135
9–<10 years	61	68	72	77	81	87	97	108	113	117	123	127	133
10–<11 years	59	66	69	75	79	85	95	106	111	115	121	125	132
11–<12 years	56	63	67	72	76	82	93	103	109	113	119	122	130
12–<13 years	53	60	64	70	74	80	90	101	107	111	116	120	127
13–<14 years	51	58	62	68	72	77	88	99	104	108	114	118	125
14–<15 years	50	57	61	66	70	76	86	97	102	106	112	115	122
15–<16 years	49	56	60	65	69	74	85	95	100	104	110	113	120

**Table 2:** Centile chart of respiratory rate per minute by age group.

Age	Centile												
	1st	3rd	5th	10th	15th	25th	50th	75th	85th	90th	95th	97th	99th
0–<3 months	20	24	26	29	31	34	39	44	47	49	52	54	58
3–<6 months	20	23	25	27	29	32	36	41	44	45	48	50	53
6–<9 months	20	22	24	26	28	30	34	38	40	42	44	46	48
9–<12 months	19	21	23	25	26	28	32	35	37	39	41	42	44
12–<15 months	19	21	22	24	25	27	30	33	35	36	38	39	41
15–<18 months	18	20	21	23	24	25	29	32	33	34	36	37	39
18–<21 months	17	19	20	22	23	24	27	30	32	33	35	36	38
21–<24 months	17	19	20	21	22	24	27	29	31	32	34	35	36
2–<3 years	16	18	18	20	21	22	25	28	29	30	32	33	34
3–<4 years	15	17	18	19	20	21	24	26	28	29	30	31	33
4–<5 years	15	16	17	19	19	21	23	26	27	28	29	30	32
5–<6 years	14	16	17	18	19	20	23	25	26	27	28	29	31
6–<7 years	14	16	16	18	19	20	22	24	26	27	28	29	30
7–<8 years	14	15	16	17	18	20	22	24	25	26	27	28	30
8–<9 years	14	15	16	17	18	19	21	24	25	26	27	28	29
9–<10 years	14	15	16	17	18	19	21	23	24	25	26	27	29
10–<11 years	14	15	16	17	18	19	21	23	24	25	26	27	28
11–<12 years	13	15	15	17	17	18	20	22	23	24	25	26	27
12–<13 years	13	15	15	16	17	18	20	22	23	24	25	25	27
13–<14 years	13	15	15	16	17	18	20	22	23	23	24	25	26
14–<15 years	13	15	15	16	17	18	20	21	22	23	24	25	26
15–<16 years	13	15	15	16	17	18	19	21	22	23	24	24	25

## References

[1] Kleinman M.E., Chameides L., Schexnayder S.M., Samson R.A., Hazinski M.F., Atkins D.L., Berg M.D., de Caen A.R., Fink E.L., Freid E.B. (2010). Part 14: Pediatric advanced life support: 2010 American Heart Association Guidelines for Cardiopulmonary Resuscitation and Emergency Cardiovascular Care. Circulation.

[2] Samuels M., Wieteska S. (2012). Advanced Paediatric Life Support: The Practical Approach.

[3] Duncan H., Hutchison J., Parshuram C.S. (2006). The Pediatric Early Warning System score: A severity of illness score to predict urgent medical need in hospitalized children. J. Crit. Care.

[4] Lee B., Kim D.K., Park J.D., Kwak Y.H. (2017). Clinical Considerations When Applying Vital Signs in Pediatric Korean Triage and Acuity Scale. J. Korean Med. Sci..

[5] Fleming S., Thompson M., Stevens R., Heneghan C., Pluddemann A., Maconochie I., Tarassenko L., Mant D. (2011). Normal ranges of heart rate and respiratory rate in children from birth to 18 years of age: A systematic review of observational studies. Lancet.

[6] O’Leary F., Hayen A., Lockie F., Peat J. (2015). Defining normal ranges and centiles for heart and respiratory rates in infants and children: A cross-sectional study of patients attending an Australian tertiary hospital paediatric emergency department. Arch. Dis. Child..

[7] Sepanski R.J., Godambe S.A., Zaritsky A.L. (2018). Pediatric vital sign distribution derived from a multi-centered emergency department database. Front. Pediatr..

[8] Bonafide C.P., Brady P.W., Keren R., Conway P.H., Marsolo K., Daymont C. (2013). Development of heart and respiratory rate percentile curves for hospitalized children. Pediatrics.

[9] Rigby R.A., Stasinopoulos D.M. (2004). Smooth centile curves for skew and kurtotic data modelled using the Box-Cox power exponential distribution. Stat. Med..

[10] Rigby R.A., Stasinopoulos D.M. (2014). Automatic smoothing parameter selection in GAMLSS with an application to centile estimation. Stat. Methods Med. Res..

[11] Indrayan A. (2014). Demystifying LMS and BCPE methods of centile estimation for growth and other health parameters. Indian Pediatr..

[12] Cole T.J., Green P.J. (1992). Smoothing reference centile curves: The LMS method and penalized likelihood. Stat. Med..

[13] Cole T.J. (1990). The LMS method for constructing normalized growth standards. Eur. J. Clin. Nutr..

[14] Davies P., Maconochie I. (2009). The relationship between body temperature, heart rate and respiratory rate in children. Emerg. Med. J..

[15] Daymont C., Bonafide C.P., Brady P.W. (2015). Heart rates in hospitalized children by age and body temperature. Pediatrics.

[16] Nijman R.G., Thompson M., van Veen M., Perera R., Moll H.A., Oostenbrink R. (2012). Derivation and validation of age and temperature specific reference values and centile charts to predict lower respiratory tract infection in children with fever: Prospective observational study. Br. Med. J..

[17] Thompson M., Harnden A., Perera R., Mayon-White R., Smith L., McLeod D., Mant D. (2009). Deriving temperature and age appropriate heart rate centiles for children with acute infections. Arch. Dis. Child..

